# Safety, Antitumor Activity, and Pharmacokinetics of Toripalimab, a Programmed Cell Death 1 Inhibitor, in Patients With Advanced Non–Small Cell Lung Cancer: A Phase 1 Trial

**DOI:** 10.1001/jamanetworkopen.2020.13770

**Published:** 2020-10-05

**Authors:** Zhijie Wang, Jianming Ying, Jiachen Xu, Pei Yuan, Jianchun Duan, Hua Bai, Changyuan Guo, Lin Li, Zhenlin Yang, Rui Wan, Kailun Fei, Zhe Zhao, Xinyang Du, Junhui Zhao, Ning Lv, Jie Wang

**Affiliations:** 1 Department of Medical Oncology, State Key Laboratory of Molecular Oncology, National Cancer Center, National Clinical Research Center for Cancer, Cancer Hospital, Chinese Academy of Medical Sciences and Peking Union Medical College, Beijing, People’s Republic of China; 2 Department of Pathology, National Cancer Center, National Clinical Research Center for Cancer, Cancer Hospital, Chinese Academy of Medical Sciences and Peking Union Medical College, Beijing, People’s Republic of China; 3 Department of Thoracic Surgery, National Cancer Center, National Clinical Research Center for Cancer, Cancer Hospital, Chinese Academy of Medical Sciences and Peking Union Medical College, Beijing, China; 4 Department of Medical Oncology, Affiliated Hospital of Qinghai University, Qinghai, China

## Abstract

**Importance:**

Programmed cell death 1 (PD-1) antibodies have shown substantial survival benefit in patients with advanced non–small cell lung cancer (NSCLC). Toripalimab is a promising and practicable PD-1 antibody; however, its performance in NSCLC has not been established.

**Objectives:**

To assess the safety, antitumor activity, and pharmacokinetics of toripalimab in patients with advanced NSCLC and to evaluate the utility of JS311, a novel PD ligand 1 (PD-L1) immunohistochemistry (IHC) assay.

**Design, Setting, and Participants:**

This single-arm open-label phase 1 trial enrolled 41 patients with advanced NSCLC that had progressed after at least 3 lines of therapy between September 21, 2017, and June 5, 2018, with a median (interquartile range) follow-up of 14.9 (3.2-22.5) months and included a cohort study comparing JS311 with other PD-L1 IHC assays that included 280 NSCLC specimens collected from January 1, 2016, to May 21, 2018. Data collection was conducted from September 21, 2017, to September 27, 2019, and analysis was conducted from September 27, 2019, to December 30, 2019.

**Exposure:**

Enrolled patients were administered a single dose of toripalimab, under 2 manufacturing processes and scales (200 L and 500 L), for safety and pharmacokinetic analysis within 28 days, followed by subsequent multidose infusions every 2 weeks. PD-L1 expression was determined by IHC with JS311, comparing its results with results from 22C3, 28-8, and SP263 simultaneously.

**Main Outcomes and Measures:**

Progression-free survival (PFS) and overall survival (OS), estimated by Kaplan-Meier curves, and continuous variables compared by *t* test or Mann-Whitney test. Correlations between PD-L1 IHC antibodies were evaluated by Spearman correlation test.

**Results:**

A total of 41 patients (29 [70.7%] men) with a median (interquartile range) age of 59 (53 to 63) years who experienced disease progression following chemotherapy were included. The most common treatment-related adverse events were rash (6 [14.6%]), increased amylase level (5 [12.2%]), and increased aspartate aminotransferase level (5 [12.2%]). In 35 patients included in the pharmacokinetic analysis, drug exposure and area under curve after 1 dose was similar under both manufacturing processes and scales (mean [SD] for 200-L group: 12 465.28 [4128.17] hour × μg/mL; for 500-L group: 12 331.42 [2472.58] hour × μg/ml). In 28 patients included in the response and survival analysis, the median PFS and OS were 2.8 (95% CI, 2.7 to 4.6) months and 13.8 months (95% CI, 10.0 months to not reached), respectively. Stratified by PD-L1 tumor proportion score of at least 50%, 1% to 49%, and less 1%, median PFS rates were 11.2 months (95% CI, 2.3 months to not evaluable), 2.3 (95% CI, 1.7 to 2.7) months, and 2.8 (95% CI, 2.7 to 4.6) months, respectively. A total of 4 anti–PD-L1 IHC antibodies were compared during PD-L1 staining, using 280 NSCLC specimens. The consistency rates between the 4 antibodies were 80.8% to 89.5% (ρ, 0.619 to 0.790) and 93.3% to 95.5% (ρ, 0.691 to 0.773), with PD-L1 tumor proportion scores of 1% and 50% as cut points, respectively.

**Conclusions and Relevance:**

In this study, toripalimab exhibited encouraging antitumor activity and manageable safety profiles among patients with heavily treated NSCLC. The novel PD-L1 IHC antibody JS311 was highly consistent with previously verified PD-L1 IHC assays.

## Key Points

**Question:**

What is the performance of toripalimab, a programmed cell death 1 (PD-1) antibody, and JS311, a novel PD ligand 1 (PD-L1) immunohistochemistry assay, among patients with non–small cell lung cancer?

**Findings:**

In this phase 1 trial that enrolled 41 patients with advanced non–small cell lung cancer, toripalimab exhibited encouraging antitumor activity and manageable safety, with median progression-free survival of 11.2, 2.3, and 2.8 months, stratified by PD-L1 tumor proportion scores of at least 50%, 1% to 49%, and less than 1%, respectively. In a cohort of 280 specimens from patients with non–small cell lung cancer, JS311 was highly consistent with previously verified PD-L1 assays.

**Meaning:**

In this study, toripalimab and JS311 exhibited potential utility in future clinical practice for patients with non–small cell lung cancer.

## Teaser

This phase 1 trial assesses the safety, antitumor activity, and pharmacokinetics of toripalimab in patients with advanced non–small cell lung cancer (NSCLC) and evaluates the utility of JS311, a novel programmed death cell ligand 1 immunohistochemistry assay.

## Introduction

Non–small cell lung cancer (NSCLC) is the leading cause of cancer-related death worldwide.^1^ Immune checkpoint inhibitors (ICIs), such as programmed cell death 1 (PD-1) and PD ligand 1 (PD-L1) monoclonal antibodies, have shown enormous survival benefits among patients with advanced NSCLC, especially those without driver variants.^2,3,4,5^ Accumulating evidence supports the clinical application of anti-PD-1/PD-L1 treatment in either first-line or salvage settings in patients with NSCLC as monotherapy or with chemotherapy,^6,7,8,9,10,11,12,13,14,15,16^ and novel agents are continually under investigation. Toripalimab is a novel humanized immunoglobin G4 monoclonal antibody against PD-1.^17^ Several phase 1/2 clinical trials of toripalimab have exhibited its manageable safety profile and promising antitumor activity among patients with advanced melanoma, urothelial cancer, renal cell cancer, and advanced gastric cancer.^18,19^ However, the performance of toripalimab in patients with NSCLC has not been established.

Several studies have demonstrated a positive correlation between PD-L1 expression with the efficacy of ICIs, although a small subset of patients with negative PD-L1 expression also obtained benefit.^6,7,8,9,10,11,12^ PD-L1 expression of at least 50% or at least 1% yielded superior or noninferior survival outcomes compared with standard platinum-based doublet chemotherapy, respectively, highlighting the importance of PD-L1 expression testing in guiding ICIs in clinical practice and improving drug and companion diagnostic codevelopment.^6,7,8^ To date, almost all US Food and Drug Administration–approved anti-PD-1/PD-L1 agents use their coupled PD-L1 immunohistochemical (IHC) assays for clinical application. For example, pembrolizumab is assessed by 22C3 antibody (Dako)^2,20^; nivolumab by 28-8 (Dako)^21^; atezolizumab by SP142 (Ventana Medical Systems)^10^; and durvalumab by SP263 (Ventana Medical Systems).^22,23^ The Blueprint PD-L1 IHC Assay Comparison Project revealed that the sensitivity of PD-L1 staining on tumor cells by 22C3, 28-8, and SP263 were comparable, whereas the SP142 assay exhibited a weaker staining,^24^ which was also observed in another study.^25^

For toripalimab, preliminary analyses demonstrated significant correlations between higher PD-L1 expression and better response and prognosis. As assessed by SP142, Tang et al^18^ reported that patients with PD-L1 expression of at least 50% have the most favorable objective response rate (ORR) and disease control rate (DCR) as well as superior overall survival (OS) in metastatic melanoma and urological cancers.^18^ Similarly, Wang et al^19^ demonstrated that patients with PD-L1 expression of at least 1% have significantly better ORR and DCR in advanced gastric cancer.^19^ JS311 is a novel PD-L1 IHC antibody staining mainly membrane and cytoplasma, which was developed specifically to guide the application of toripalimab. However, it is unknown whether JS311 can differentiate the response to toripalimab or be comparable with other commercial PD-L1 IHC staining antibodies.

In this study, we investigated the safety and antitumor activity of toripalimab in advanced NSCLC based on a phase 1 trial, which evaluated the pharmacokinetics of toripalimab under 2 manufacturing processes and scales (200 L vs 500 L). The utility and comparability of JS311 in PD-L1 IHC staining were examined by analyzing the consistency with 3 other commonly used antibodies in specimens from 280 patients with NSCLC.

## Methods

### Study Design and Participants

This was a single-center, open-label, parallel-control, phase 1 trial (NCT03301688) to compare the pharmacokinetics, safety, and antitumor activity of toripalimab manufactured under 2 processes and scales in patients with advanced NSCLC. Eligibility criteria included being aged 18 to 75 years; having an Eastern Cooperative Oncology Group score of 0 or 1; having histologically confirmed and standard treatment–recurrent or standard treatment–intolerant stage IV NSCLC; being enrolled between September 21, 2017, and June 5, 2018; and being successively assigned to 2 groups that received toripalimab at a fixed dose of 3 mg/kg via intravenous infusion under 200 L or 500 L manufacturing processes. This study was approved by the ethics committees of the Cancer Hospital, Chinese Academy of Medical Sciences, and conducted in accordance with the Declaration of Helsinki^26^ and the international standards of good clinical practice. All enrolled patients provided written informed consent. This study followed the Consolidated Standards of Reporting Trials (CONSORT) reporting guideline.

This study consisted of an initial single-dose phase and subsequent multidose phase. During the single-dose phase, patients were given 1 dose of toripalimab and monitored for safety and pharmacokinetics profiles for 28 days. If the participant did not experience adverse events with clinical significance and could possibly benefit from continuous treatment as assessed by investigators, they could advance to the subsequent multidose phase after giving consent. During the multidose phase, patients were treated with toripalimab every 2 weeks until disease progression, intolerable adverse event(s), voluntary withdrawal, or the development of unsuitable physical condition to receive treatment as evaluated by investigators. During the multidose phase, the safety and antitumor activity of toripalimab and survival were analyzed.

To compare JS311 with other PD-L1 IHC antibodies, we collected biopsy specimens from 280 patients with NSCLC receiving care in Cancer Hospital, Chinese Academy of Medical Sciences, from January 2016 to May 2018, including 168 cases with lung adenocarcinoma and 112 cases with lung squamous cell carcinoma.

### Treatment Evaluation

Safety assessments were conducted for all patients at baseline and regular intervals. The severity of all adverse events was graded based on National Cancer Institute Common Terminology Criteria for Adverse Events version 4.0. Severe adverse events were defined as any event leading to death, life-threatening or prolonged hospitalization, severe or permanent deformity or dysfunction, or other critical adverse event deteriorating the disease.

Antitumor activity was assessed via radiological evaluations by computed tomography and magnetic resonance imaging every 8 weeks during the multidose phase using Response Evaluation Criteria in Solid Tumors version 1.1. The response evaluation is detailed in the eAppendix in the Supplement. The follow-up was from September 21, 2017, to September 27, 2019.

### Pharmacokinetics

Serum samples were collected for pharmacokinetics analysis. More details appear in the eAppendix in the Supplement.

### PD-L1 Expression Analysis in Tumor Biopsies

PD-L1 expression analysis was performed using 22C3 (Dako), 28-8 (Abcam), SP263 (Ventana), and JS311 (Junshi) in tumor biopsies and was scored by membranous tumor proportion score (TPS), as detailed in the eAppendix in the Supplement. IHC results were examined by 2 masked independent pathologists (J.Y. and P.Y.). All inconsistencies were reviewed and confirmed by the pathological review board (J.Y., P.Y., and N.L.).

### Genomic Profiling

DNA sequencing was performed for formalin-fixed, paraffin-embedded tumor biopsies and paired blood samples. Details appear in the eAppendix in the Supplement.

### Indirect Immunofluorescence Staining

A549, H157, GLC82, H1299, and PC9 were subjected to indirect immunofluorescence staining with 4 PD-L1 primary antibodies to identify PD-L1 expression in the tumor biopsy. Details appear in the eAppendix in the Supplement.

### Cell Labeling and Fluorescence Activated Cell Sorting

Flow cytometry analysis was performed with fluorescein-labeled 4 PD-L1 antibodies. Details appear in the eAppendix in the Supplement.

### Statistical Analysis

The sample size was calculated based on the pharmacokinetic parameter drug exposure and area under curve (AUC_0-t_). Assuming that the AUC coefficient of variation was 20% and its geometric mean ratio (under 2 manufacturing processes, ie, 200 L vs 500 L) ranged from 0.95 to 1.05, enrollment of at least 30 participants (15 in each treatment group) could provide more than 80% power showing that the geometric mean ratio’s 80% CI for AUC would fall within the range of 80% and 125%. The Kaplan-Meier method was used to estimate PFS and OS, and the log-rank test was used to compare the difference between curves. Continuous variables were compared by *t* test or Mann-Whitney test, and categorical variables were compared by Fisher exact test. Correlations between PD-L1 primary antibodies were evaluated by the Spearman correlation test, and a high correlation was defined as ρ of at least 0.70. A 2-tailed *P* < .05 was considered statistically significant. SPSS statistical software version 22 (IBM Corp) and Prism version 8.0 (GraphPad) were used for all analyses.

## Results

### Clinical Characteristics

Overall, 41 patients (29 [70.7%] men and 12 [29.3%] women, with a median [interquartile range] age of 59 [53-63] years) with advanced NSCLC sequentially received toripalimab under 200 L (group A, 20 patients [48.8%]) or 500 L (group B, 21 patients [51.2%]) manufacturing processes. The baseline clinicopathological characteristics were similar between the groups (eTable 1 in the Supplement). Overall, 14 (34.1%) and 20 (48.8%) patients had received prior surgery and radiotherapy, respectively, and all patients had received prior chemotherapy for metastatic disease. The median (range) lines of prior treatments were 2 (1-7), and 10 patients (24.4%) were heavily pretreated, with at least 3 lines of therapy. The flow chart of participants is shown in eFigure 1 in the Supplement.

### Pharmacokinetics

A total of 35 patients completed the intended blood collection points for pharmacokinetic analysis. The pharmacokinetic parameters are listed in eTable 2 in the Supplement. The primary end point, drug exposure and AUC_0-t_ after 1 dose was a mean (SD) of 12 465.28 (4128.17) hour × μg/mL for group A and 12 331.42 (2472.58) hour × μg/ml for group B (*P* = .91) (eFigure 2 in the Supplement). Most pharmacokinetic parameters, such as the peak concentration, time to peak, half time, and so on, were similar between the 2 groups. The relative bioavailability of group B was 98.9% compared with group A.

### Safety

At the cutoff date (ie, September 27, 2019), 18 patients (43.9%) had experienced at least 1 treatment-related adverse event, including 11 (55.0%) in group A and 9 (42.8%) in group B (Table). The spectra and frequencies of treatment-related adverse events were similar between the 2 groups. The most common treatment-related adverse events were rash (6 of 41 [14.6%]), increased amylase level (5 [12.2%]), and increased aspartate aminotransferase level (5 [12.2%]). Grade 3 to 4 treatment-related adverse events were observed in 1 patient (2.4%) in group B. A total of 3 patients (15.0%) in group A and 5 (23.8%) in group B experienced severe adverse events (eTable 3 in the Supplement), most of which were probably unrelated to treatment, except for 2 patients who experienced interstitial lung disease. No treatment-related deaths were reported.

**Table.** Treatment-Related Adverse Events

Event	Patients, No. (%)		
	Group A (n = 20)	Group B (n = 21)	Total (N = 41)
Any adverse event	11 (55.0)	9 (42.8)	18 (43.9)
≥Grade 3 adverse event	0	1 (4.8)	1 (2.4)
Rash	3 (15.0)	3 (14.3)	6 (14.6)
Amylase level increase			
Any	3 (15.0)	2 (9.5)	5 (12.2)
≥Grade 3	0	1 (4.8)	1 (2.4)
Aminotransferase level increase			
Aspartate	1 (5.0)	4 (19.0)	5 (12.2)
Alanine	1 (5.0)	2 (9.5)	3 (7.3)
γ-Glutamyltransferase level increase	1 (5.0)	2 (9.5)	3 (7.3)
Hypothyroidism	1 (5.0)	2 (9.5)	3 (7.3)
Hyperthyroidism	0	2 (9.5)	2 (4.9)
Subclinical hypothyroidism	0	2 (9.5)	2 (4.9)
Nausea	2 (10.0)	0	2 (4.9)
Blood bilirubin increased	0	2 (9.5)	2 (4.9)
Conjugated blood bilirubin increased	0	2 (9.5)	2 (4.9)
Unconjugated blood bilirubin increased	0	1 (4.8)	1 (2.4)
White blood cell count decreased	1 (5.0)	0	1 (2.4)
Neutrophil count increased	1 (5.0)	0	1 (2.4)
Blood lipase elevated	0	1 (4.8)	1 (2.4)
Any	0	1 (4.8)	1 (2.4)
≥Grade 3	0	1 (4.8)	1 (2.4)
Blood thyroid stimulating hormone increased	0	1 (4.8)	1 (2.4)
Lumbar pain	1 (5.0)	0	1 (2.4)
Immune-related pneumonia	1 (5.0)	0	1 (2.4)
Fatigue	1 (5.0)	0	1 (2.4)
Scalp itchiness	1 (5.0)	0	1 (2.4)
Excessive sweating	1 (5.0)	0	1 (2.4)
Toothache	1 (5.0)	0	1 (2.4)

### Antitumor Activity

**Figure 1.**
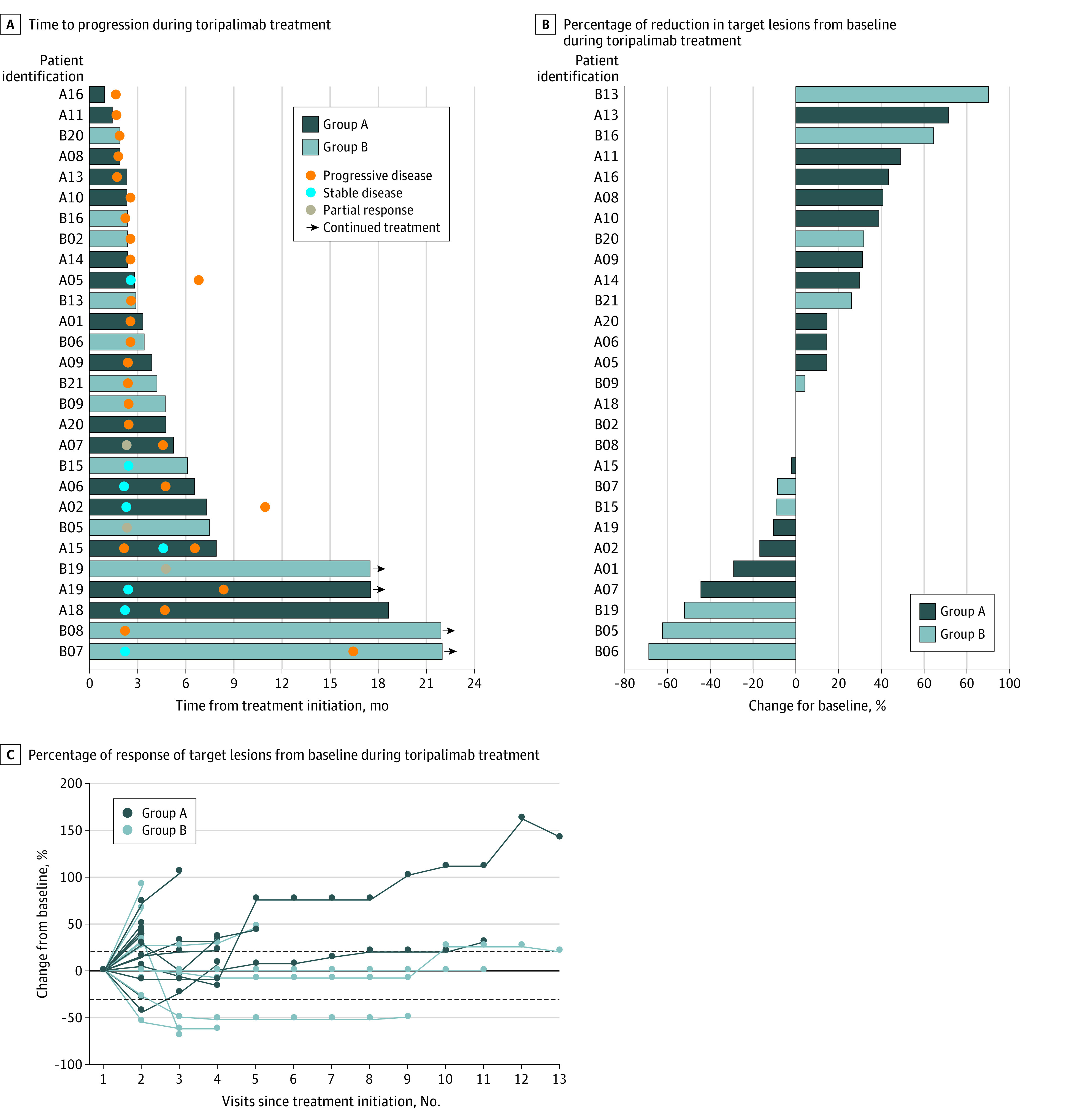
Antitumor Activity of Toripalimab in Response and Survival Analysis Set of 28 Patients

**Figure 2.**
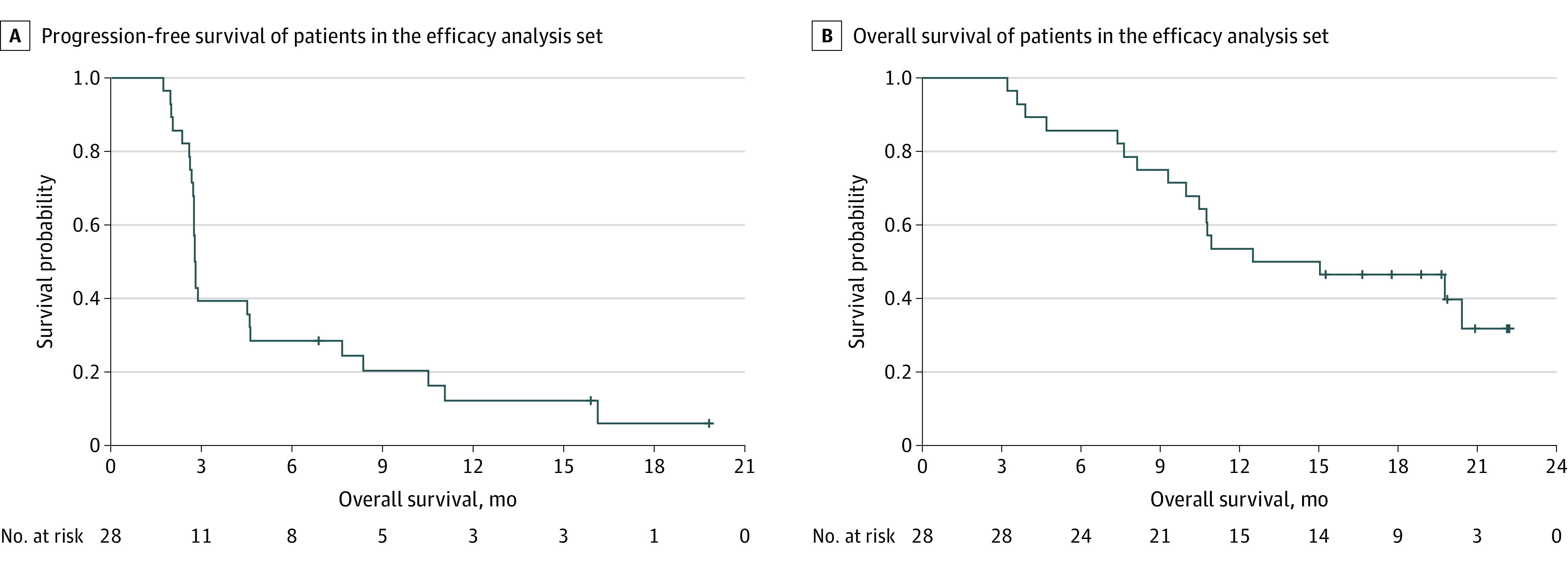
Kaplan-Meier Curves for Survival in the Response and Survival Analysis Set of 28 Patients

By the cutoff date (ie, September 27, 2019), 28 patients (16 [57.1%] in group A and 12 [42.9%] in group B) had at least 1 posttreatment evaluation and were included in the response and survival analysis (Figure 1; eTable 4 in the Supplement). The median (range) follow-up time was 14.9 (3.2 to 22.5) months. The confirmed ORR was 7.1% (2 of 28; 0 in group A and 2 [16.7%] in group B), and the DCR was 39.3% (11 of 28; 6 [37.5%] in group A and 5 [41.7%] in group B). Five patients (17.9%) were still on treatment, and 2 of them (40.0%) were under continuous disease control. The median (range) duration of treatment was 4.0 (0.9 to >22.0) months. The median PFS was 2.8 (95% CI, 2.7 to 4.6) months (group A: 2.7 [95% CI 2.6 to 4.6] months; group B, 2.8 [95% CI, 2.3 to 16.1] months) (Figure 2A; eFigure 3A in the Supplement). The 3-month and 6-month PFS rates were 39.3% (95% CI, 21.7% to 56.1%) and 28.6% (95% CI, 13.5% to 45.6%), respectively. The median OS was 13.8 months (95% CI, 10.0 months to not reached [NR]) for all patients (group A, 10.9 [95% CI, 8.1 to 20.4] months; group B, NR (95% CI, 3.6 months to NR) (Figure 2B; eFigure 3B in the Supplement). At 12 and 18 months, 53.6% (95% CI, 33.8%-69.8%) and 46.4% (95% CI, 27.6%-63.3%) of patients were alive, respectively. For the 41 patients included in the intention-to-treat analysis, median PFS and OS are displayed in eFigure 3C, eFigure 3D, eFigure 3E, and eFigure 3F in the Supplement. After disease progression or withdraw from the trial, 11 patients (26.8%), 7 patients (17.1%), and 3 patients (7.3%) received chemotherapy, oral tyrosine kinase inhibitors, and radiotherapy, respectively, and 3 patients (7.3%) continued toripalimab treatment.

### Correlation Between PD-L1 Expression by JS311 and Clinical Outcomes

Among 28 patients in the response and survival analysis, tumor tissues were available from 23 (82.1%) for PD-L1 expression assessment by JS311, including 3 (13.0%) with TPS of at least 50%, 2 (8.7%) with TPS of 1% to 49%, and 18 (78.3%) with TPS of less than 1%. The ORRs for these 3 subgroups were 33.3% (1 of 3), 0% (0 of 2) and 5.6% (1 of 18) (*P* = .26), and the DCRs were 66.7% (2 of 3), 0% (0 of 2), and 27.8% (5 of 18) (*P* = .28), respectively. The median PFS was 11.2 months (95% CI, 2.3 months to not evaluable), 2.3 (95% CI, 1.7 to 2.7) months, and 2.8 (95% CI, 2.7 to 4.6) months (*P* = .05), respectively. After excluding 5 patients harboring activated epidermal growth factor receptor variants (3 [60.0%] with Exon 21 p.L858R and 2 [40.0%] with Exon 20 insertion), 18 patients were further analyzed, including 2 (11.1%) with TPS of at least 50%, 2 (11.1%) with TPS of 1% to 49%, and 14 (77.8%) with TPS of less than 1%. The ORRs for these subgroups were 50.0% (1 of 2), 0% (0 of 2) and 7.1% (1 of 14) (*P* = .17), and the DCRs were 100.0% (2 of 2), 0% (0 of 2), and 28.6% (4 of 14) (*P* = .04), respectively. The median PFS rates were snot evaluable (95% CI, 11.0 months to not evaluable), 2.3 (95% CI, 1.7 to 2.7) months, and 2.8 (95% CI, 2.7 to 7.6) months, respectively. Notably, the 2 patients with TPS of at least 50% had a durable response and were still under treatment at the time of data cutoff. We further analyzed the blood tumor variation burden status of 5 patients with PFS of at least 3 months who had PD-L1 TPS of less than 1%; 4 (80.0%) had sufficient blood samples, and the median blood tumor variation burden was 12.5 mutations per megabase pairs.

### Pairwise Comparisons of PD-L1 IHC Staining by JS311 and Other Assays

**Figure 3.**
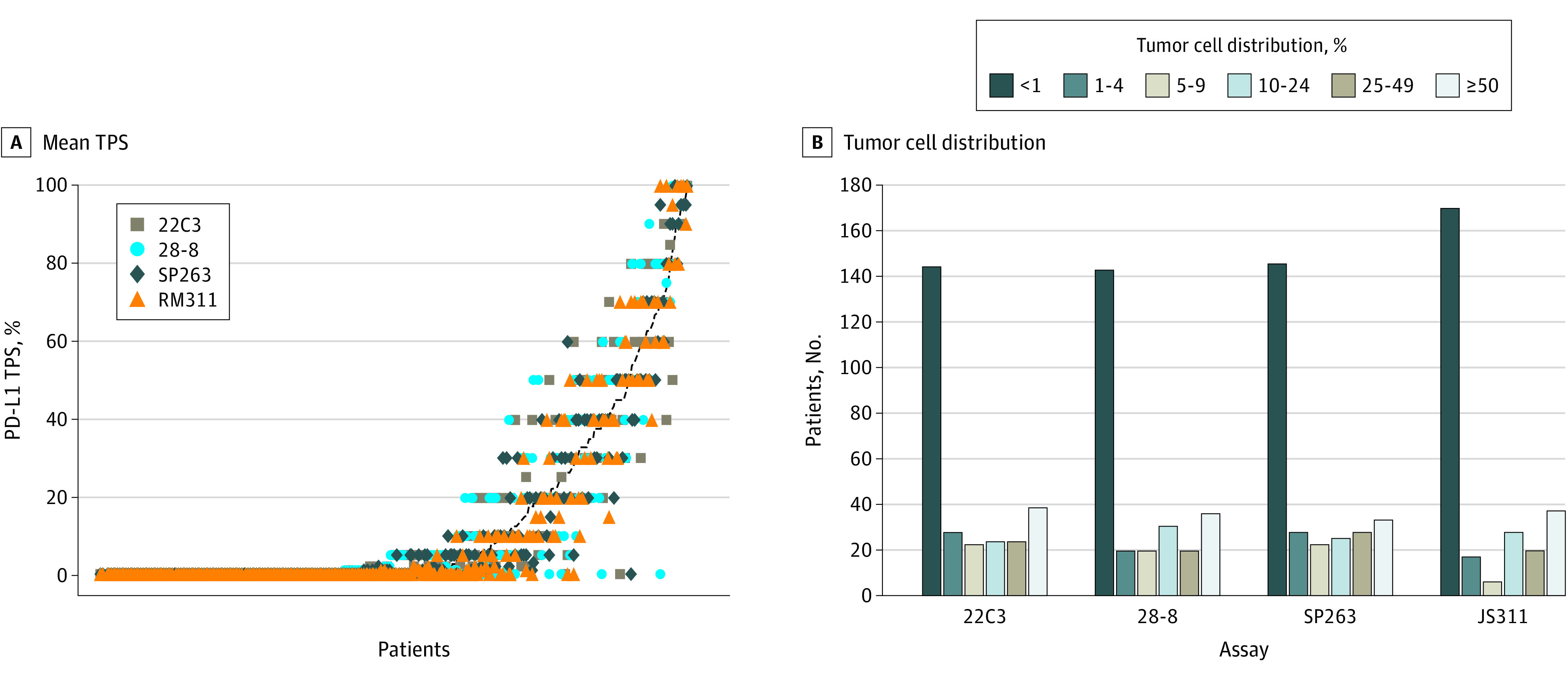
Pairwise Comparisons of Programmed Cell Death Ligand 1 (PD-L1) Staining as Defined by 22C3, 28-8, SP263, and JS311 TPS indicates tumor proportion score.

Tumor specimens from 280 patients with advanced NSCLC (eTable 5 in the Supplement) were collected for PD-L1 IHC staining using 4 anti-PD-L1 antibodies. The positive rates of 22C3, 28-8, SP263, and JS311 with a 1% TPS as a cut point were 48.2% (134 of 278), 46.6% (125 of 268), 48.0% (134 of 279), and 38.4% (106 of 276), respectively, and, with 50% TPS as the cut point, 13.7% (38 of 278), 13.4% (36 of 268), 11.8% (33 of 279) and 13.4% (37 of 276), respectively. The PD-L1 TPS and tumor cell distribution of PD-L1 expression of PD-L1 positivity of 4 antibodies is shown in Figure 3. The PD-L1 staining levels were classified in 3 categories (<1%, 1%-49%, and ≥50%), and the consistency rates of these 4 PD-L1 antibodies were 74.8% to 84.5% (ρ, 0.692-0.817) in all cases. With 1% and 50% as the cut points, the consistency rates of these antibodies were 80.8% to 89.5% (ρ, 0.619-0.790) and 93.3% to 95.5% (ρ, 0.691-0.773), respectively (eTable 6, eTable 7, and eTable 8 in the Supplement).

**Figure 4.**
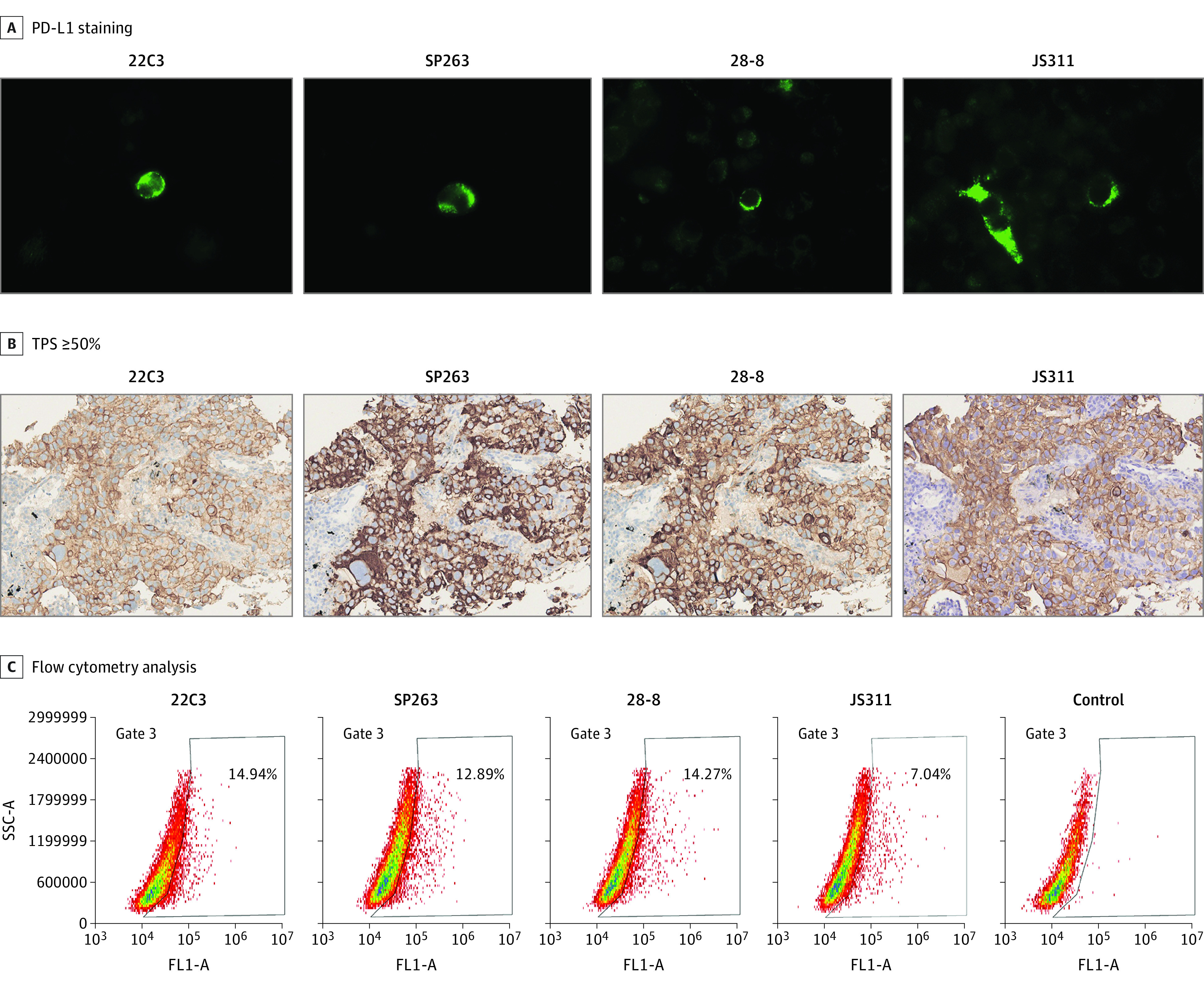
Consistency Among 4 Antibodies in A549 Cell Lines B, Images have magnification ×200. C, The flow cytometry analysis of the 4 programmed cell death ligand 1 (PD-L1) antibodies. SSC-A indicates side-scatter area; TPS, tumor proportion score.

To further reduce the potential disturbance of the bias owing to population heterogeneity and subjective interpretation, the assays of indirect immunofluorescence staining were performed using the 4 PD-L1 antibodies, confirming the high consistency across 5 lung cancer stable cell lines (ie, A549, H157, GLC82, H1299 and PC9). The consistencies were further confirmed by flow cytometry analysis (Figure 4; eFigure 4, eFigure 5, and eFigure 6 in the Supplement).

## Discussion

Anti-PD-1/PD-L1 treatment has displayed a wide spectrum of antitumor activity across various tumors, and several anti-PD-1/PD-L1 agents have demonstrated clinical efficacy and manageable tolerance in patients with NSCLC. To our knowledge, we have for the first time reported the safety, antitumor activity, and pharmacokinetics of a novel anti-PD-1 inhibitor toripalimab in patients with heavily treated advanced NSCLC. We also explored the utility of a novel PD-L1 antibody JS311 in IHC staining by comparing it with 3 other commonly used antibodies in a large NSCLC cohort.

In the present study, the pharmacokinetic parameters were similar between toripalimab from 2 manufacturing processes. Generally, the incidence of adverse events and grade 3 or 4 treatment-related adverse events were comparable with previous reports.^18,19^ In early phase 1/2 clinical trials, toripalimab exhibited its antitumor activity in patients with chemotherapy-refractory advanced malignant neoplasms.^18,19^ The median PFS and OS of patients with NSCLC treated with toripalimab in this study were 2.8 months and 13.8 months, respectively, which are consistent with previous studies of ICIs as second-line treatment for advanced NSCLC,^2,4,5,27,28^ although ORR and DCR were lower in this study. Notably, 10 patients (24.4%) were heavily pretreated, with at least 3 lines of prior treatment, and 11 (26.8%) had gene alterations that are correlated with poor response to ICI treatment, such as activated *EGFR* variants,^5,29,30,31,32^ *ALK* amplification,^30^ *JAK* loss,^33^ and *DNMT3A* variants.^34^ Only approximately 20% of the enrolled patients exhibited positive PD-L1 expression, lower than the reported prevalence of PD-L1 expression in NSCLC.^35^ These indices might partially explain why our study presented inferior ORR. Notably, by the time of data collection, 5 patients (17.9%) remained on treatment. In general, toripalimab displayed promising antitumor activity with a favorable toxicity profile in patients with heavily pretreated NSCLC, which warrants further evaluation.

Positive PD-L1 expression was an inclusion criteria in several ICI clinical trials owing to its correlation with better outcomes. Currently, the commonly used cut point of PD-L1 expression on tumor cells are 1% and 50%. In our study, the ORR of the subgroup with PD-L1 expression of at least 50% subgroup was 33.3%, which is comparable to that in KEYNOTE-024,^7^ KEYNOTE-042,^8^ and CheckMate 017/057.^4,5^ After excluding patients with *EGFR* variants, more distinct differences could be observed among different PD-L1 expression subgroups, suggesting the potentially positive correlation between PD-L1 expression assessed by JS311 and the clinical outcomes of toripalimab; however, more prospective cohorts with larger sample sizes are needed.

Several previous studies have assessed the concordance among various PD-L1 assays. A consistently high concordance of 22C3, 28-8, and SP263 has been demonstrated in different studies, with correlation coefficients between 0.753 and 0.909,^36^ between 0.80 and 092,^37^ and between 0.726 and 0.812,^38^ suggesting that PD-L1 expression variations in different trials were independent of the different assays they used, and each assay was sufficient to serve as the basis of therapeutic decision regarding anti-PD-1/PD-L1 treatment. In our study, we demonstrated a good concordance between these 3 antibodies with JS311, regardless of PD-L1 cut point (ie, 1% or 50%). Currently, the selection criteria for first-line and second-line pembrolizumab is PD-L1 expression of at least 50% and at least 1% by 22C3, respectively. Given the comparable results, JS311 might be applied to guide toripalimab treatment in future clinical practice.

### Limitations

This study has limitations. The small sample size and the unselected enrollment of patients might result in statistical bias and limit the statistical power of this study to some degree. The ORR of group B was higher than that of group A; however, all other clinical outcomes, including DCR, PFS, and safety profiles and pharmacokinetics, were very similar between group A and group B. As such, the observed intergroup difference of ORR might be attributed to the relatively limited sample size and single-group design. Generally, altered manufacturing processes did not reduce the antitumor activity. Moreover, the PD-L1 expression results were obtained through 2 pathologists but may need to be confirmed by additional pathologists in a double-masked setting. In addition, the value of antibody JS311 to differentiate the response to toripalimab needs to be verified in a larger cohort treated by ICIs.

## Conclusions

This phase 1 trial demonstrated the encouraging antitumor activity and a manageable safety profile of toripalimab in patients with advanced NSCLC treated with at least 3 lines of therapy. Promising long-term survival and a durable response were observed in patients with PD-L1 expression of at least 50%, as assessed by novel PD-L1 antibody JS311. PD-L1 expression assessed by JS311 was highly consistent with 3 commonly used and previously verified PD-L1 staining assays. These results can contribute to the diversity of anti–PD-1/PD-L1 treatment and provide an interchangeable assay for pathological practice.

## Notes

Supplement. **eAppendix. **Supplementary Methods **eFigure 1. **Study Flow Chart **eFigure 2. **Peak Concentration (C_max_) and Drug Exposure (AUC_0 − t_) of JS311 Under Old and New Pharmaceutical Processes **eFigure 3. **Kaplan-Meier Curves for Survival **eFigure 4. **Consistency Among 4 Antibodies in A549 Cell Lines **eFigure 5. **Flow Cytometry Analysis of 4 Antibodies in 5 Cell Lines **eFigure 6. **Indirect Immunofluorescence Staining Using 4 Antibodies in 5 Cell Lines **eTable 1. **Baseline Clinicopathological Characteristics **eTable 2. **Pharmacokinetic Parameters of Patients **eTable 3. **Summary of Severe Adverse Events **eTable 4. **Best Response According to RECIST **eTable 5. **Baseline Clinicopathological Characteristics of Assay Cohort **eTable 6. **Consistency Rate Among 4 Antibodies **eTable 7. **Consistency Rate Among 4 Antibodies in Lung Adenocarcinoma **eTable 8. **Consistent Rate Among 4 Antibodies in Squamous Cell Carcinoma **eReferences.**
