# Supplementary material for: Safety, Antitumor Activity, and Pharmacokinetics of Toripalimab, a Programmed Cell Death 1 Inhibitor, in Patients With Advanced Non–Small Cell Lung Cancer: A Phase 1 Trial
Source: JAMA Netw Open. 2020 Oct 5;3(10):e2013770. doi: 10.1001/jamanetworkopen.2020.13770 (PMC7536589; doi:10.1001/jamanetworkopen.2020.13770)
Supplement: Supplement. — eAppendix. Supplementary Methods eFigure 1. Study Flow Chart eFigure 2. Peak Concentration (Cmax) and Drug Exposure (AUC0 − t) of JS311 Under Old and New Pharmaceutical Processes eFigure 3. Kaplan-Meier Curves for Survival eFigure 4. Consistency Among 4 Antibodies in A549 Cell Lines eFigure 5. Flow Cytometry Analysis of 4 Antibodies in 5 Cell Lines eFigure 6. Indirect Immunofluorescence Staining Using 4 Antibodies in 5 Cell Lines eTable 1. Baseline Clinicopathological Characteristics eTable 2. Pharmacokinetic Parameters of Patients eTable 3. Summary of Severe Adverse Events eTable 4. Best Response According to RECIST eTable 5. Baseline Clinicopathological Characteristics of Assay Cohort eTable 6. Consistency Rate Among 4 Antibodies eTable 7. Consistency Rate Among 4 Antibodies in Lung Adenocarcinoma eTable 8. Consistent Rate Among 4 Antibodies in Squamous Cell Carcinoma eReferences. [file jamanetwopen-e2013770-s001.pdf]

Supplemental Online Content

Wang Z, Ying J, Xu J, et al. Safety, antitumor activity, and pharmacokinetics of toripalimab, a programmed cell death 1 inhibitor, in patients with advanced non–small cell lung cancer: a phase 1 trial. *JAMA Netw Open*. 2020;3(10):e2013770. doi:10.1001/jamanetworkopen.2020.13770

eAppendix. Supplementary Methods
eFigure 1. Study Flow Chart
eFigure 2. Peak Concentration (C_{max}) and Drug Exposure (AUC_{0-t}) of JS311 Under Old and New Pharmaceutical Processes
eFigure 3. Kaplan-Meier Curves for Survival
eFigure 4. Consistency Among 4 Antibodies in A549 Cell Lines
eFigure 5. Flow Cytometry Analysis of 4 Antibodies in 5 Cell Lines
eFigure 6. Indirect Immunofluorescence Staining Using 4 Antibodies in 5 Cell Lines
eTable 1. Baseline Clinicopathological Characteristics
eTable 2. Pharmacokinetic Parameters of Patients
eTable 3. Summary of Severe Adverse Events
eTable 4. Best Response According to RECIST
eTable 5. Baseline Clinicopathological Characteristics of Assay Cohort
eTable 6. Consistency Rate Among 4 Antibodies
eTable 7. Consistency Rate Among 4 Antibodies in Lung Adenocarcinoma
eTable 8. Consistent Rate Among 4 Antibodies in Squamous Cell Carcinoma
eReferences.

This supplemental material has been provided by the authors to give readers additional information about their work.

© 2020 Wang Z et al. *JAMA Network Open.*

eAppendix. Supplementary Methods

Treatment evaluation

Antitumor activity was assessed via radiological evaluations by CT and MRI every eight weeks during the multi-dose phase using RECIST version 1.1. Objective response rate (ORR) was defined as the proportion of patients who experienced complete response (CR) or partial response (PR), as assessed by independent radiologists. Disease control rate (DCR) was defined as the proportion of patients with CR, PR or stable disease (SD). Survival was followed up every three months after progression disease (PD) or end of treatment. Progression-free-survival (PFS) was defined as the interval between randomization to PD or death. Duration of response (DOR) was defined as the interval between the first documented response (CR or PR) and the first documented PD or death. The follow-up was from September 21st 2017 to September 27th, 2019.

Pharmacokinetics

Serum samples (0.5 mL) were collected within -0.5 h (0 h) prior to treatment, immediately after administration (±5 min), 0.5 h (±5 min), 2 h (±5 min), 6 h (±5 min), 12 h (±5 min), 24 h (±30 min), 48 h (±30 min), 96 h (±30 min), 168 h (±30 min), 336 h (±30 min), 504 h (±30 min), 648 h (±30 min) from dose initiation during the single-dose phase. The non-compartment model adopting WinNonlin v6.4 software (Pharsight Inc.) was utilized for PK studies.

PD-L1 expression analysis in tumor biopsies

All biopsies were fixed with 10% formalin and embedded in paraffin (FFPE). The wax blocks were sliced continuously and underwent PD-L1 IHC staining using 22C3 (Dako), 28-8 (Abcam), SP263 (Ventana) and JS311 (Junshi). Among them, 22C3 staining was performed on Dako auto-stain system, 28-8, SP263 and JS311 used Ventana Ultraview detection system on Ventana automatic staining device. PD-L1 expression was scored by membranous tumor proportion score (TPS), the percentage of tumor cells demonstrating partial or complete

© 2020 Wang Z et al. *JAMA Network Open.*

membranous PD-L1 expression. IHC results were examined by two blinded independent pathologists (J. Ying and P. yuan). All inconsistencies were reviewed and confirmed by the Pathological Review Board (N. Lv, J. Ying, C. Guo and P. Yuan).

Genomic profiling

Genomic DNA (gDNA) from FFPE tumor biopsies and paired blood samples were isolated using the DNeasy Blood & Tissue Kit (Qiagen, Hilden, Germany). DNA sequencing was performed via a 1021-gene panel. Single nucleotide variants (SNVs) were determined using MuTect (version 1.1.4) and NChot softwares. Small insertions and deletions (indels) were called by GATK. CONTRA (v2.0.8) was used to determine somatic copy-number alternation. All candidate variants were manually verifed with the integrative genomics viewer browser.

Indirect immunofluorescence staining

A549, H157, GLC82, H1299 and PC9 were subjected to indirect immunofluorescence staining with PD-L1 primary antibodies (100 μg/mL, dilution 1:100), including 22C3 (Darko), SP263 (Ventana), 28-8 (Agilent), and JS311 (Junshi), and then incubated with Alexa-488 labelled secondary antibodies[1] to identify PD-L1 expression in the tumor biopsy. Nuclei were stained with 0.5 μg/mL 4,6-diamidino-2-phenylindole dihydrochloride (DAPI, Polysciences, Warrington, PA, USA). All specimens were mounted in 90% glycerol/phosphate-buffered saline with 2.5% 1,4-diazabicyclo (2, 2, 2) octane and assayed by confocal microscopy (SP7, Leica, Wetzlar, Germany).

Cell labeling, fluorescence activated cell sorting

For flow cytometry analysis, four PD-L1 antibodies were labeled with fluorescein using the Lightning Conjugation Kit according to the manufactuer's protocol (Cat 7400-010, Innova Biosciences Ltd., Cambridge, UK).[2] FITC-labeled antibodies (100 ng/ml) were used to stain

© 2020 Wang Z et al. *JAMA Network Open.*

lung cancer cells and were incubated in dark at 4°C for 20 mins. The labeling cells were washed, filtered and analyzed by flow cytometry (BD FACSAriaTM Cell Sorter, BD Biosciences, San Jose, CA, USA).

© 2020 Wang Z et al. *JAMA Network Open.*

eFigure 1. Study Flow Chart

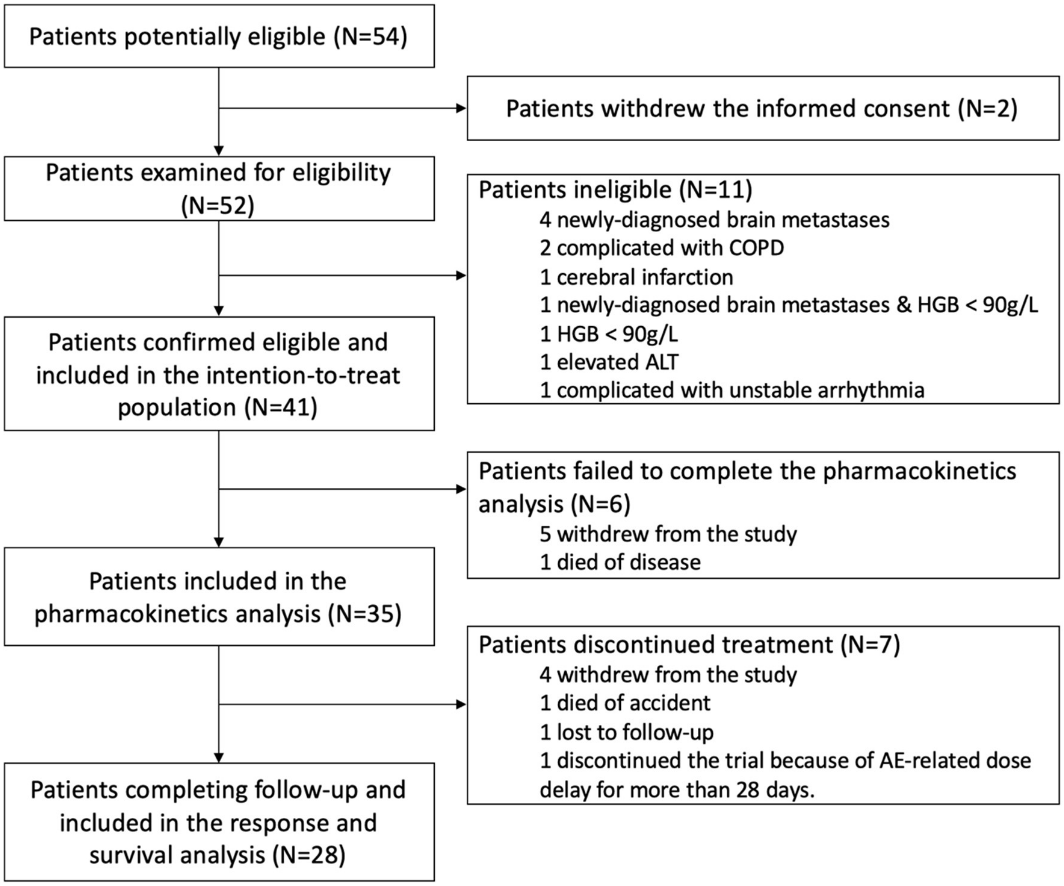

COPD = Chronic obstructive pulmonary disease. HGB = hemoglobin. ALT = Alanine aminotransferase. AE = Adverse Event.

© 2020 Wang Z et al. *JAMA Network Open.*

eFigure 2. Peak Concentration (C_{max}) and Drug Exposure (AUC_{0-t}) of JS311 Under Old and New Pharmaceutical Processes

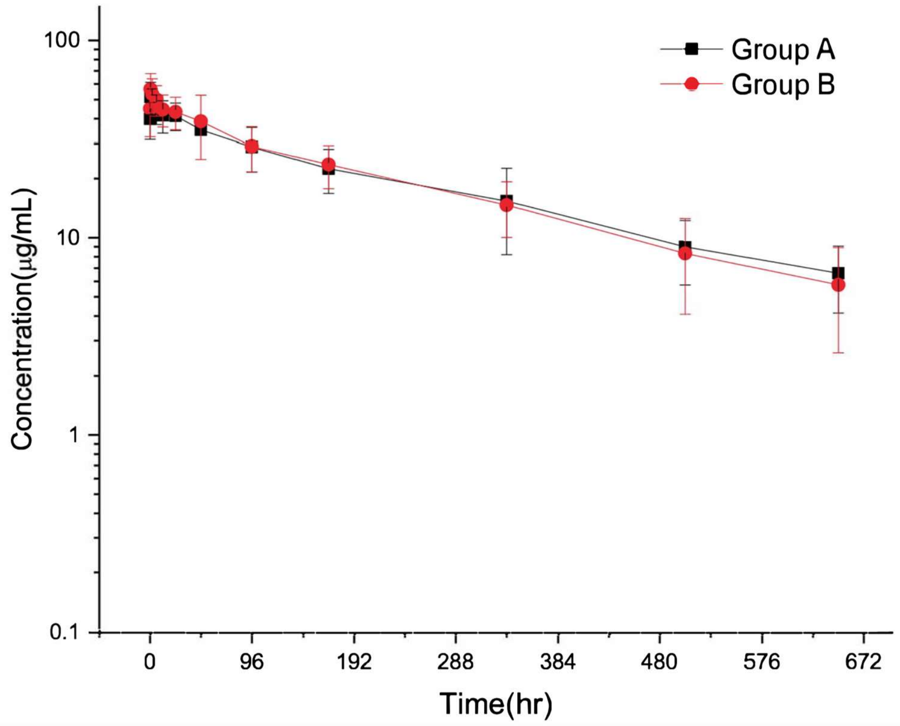

© 2020 Wang Z et al. *JAMA Network Open.*

eFigure 3. Kaplan-Meier Curves for Survival

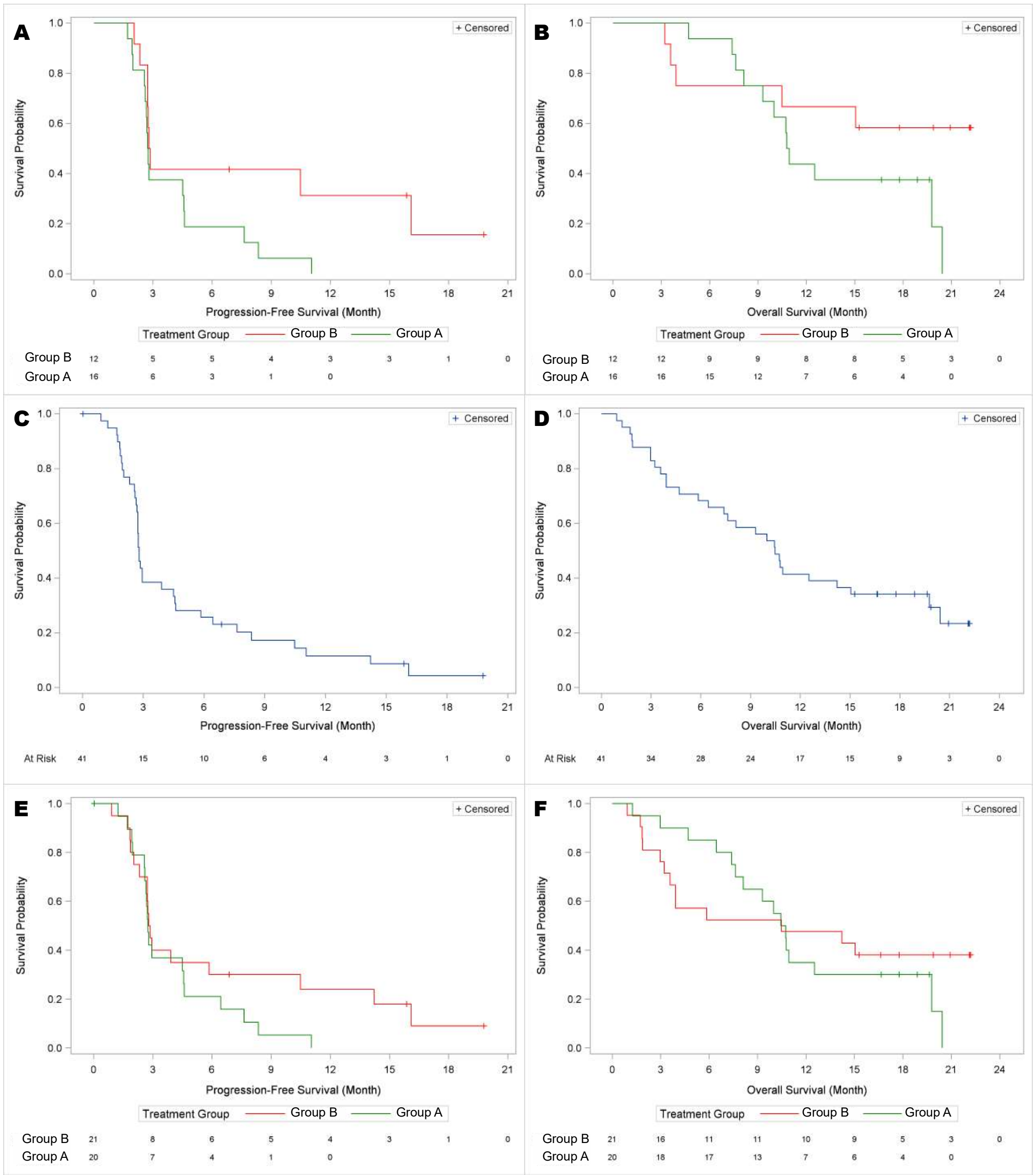

eFigure 3. Kaplan-Meier Curves for Survival

(A) The progression-free survival (PFS) of patients stratified by Group A and Group B in the response and survival analysis set (N=28). [Group A, 2.7 months (95%CI 2.6-4.6) and Group B, 2.8 months (95%CI 2.3-16.1)].

(B) The overall survival (OS) of patients stratified by Group A and Group B in the response and survival analysis set (N=28). [Group A, 2.7 months (95%CI 2.6-4.6) and Group B, 2.8 months (95%CI 2.3-16.1)].

© 2020 Wang Z et al. *JAMA Network Open.*

(C) The PFS of intention-to-treat (ITT) patients in the full analysis set (N=41). The median PFS was 2.8 months (95%CI 2.7-4.5 months).
(D) The OS of ITT patients in the full analysis set (N=41). The median OS was 10.5 months (95%CI 6.4-15.0 months).
(E) The PFS of ITT patients stratified by Group A and Group B in the full analysis set (N=41). [Group A, 2.8 months (95%CI 2.6-4.6) and Group B, 2.8 months (95%CI 2.0-10.5)].
(F) The OS of ITT patients stratified by Group A and Group B in the full analysis set (N=41). [Group A, 10.6 (95%CI 7.4-19.8) and Group B, 10.5 months (95%CI 3.2-not reached)]

© 2020 Wang Z et al. *JAMA Network Open.*

eFigure 4. Consistency Among 4 Antibodies in A549 Cell Lines

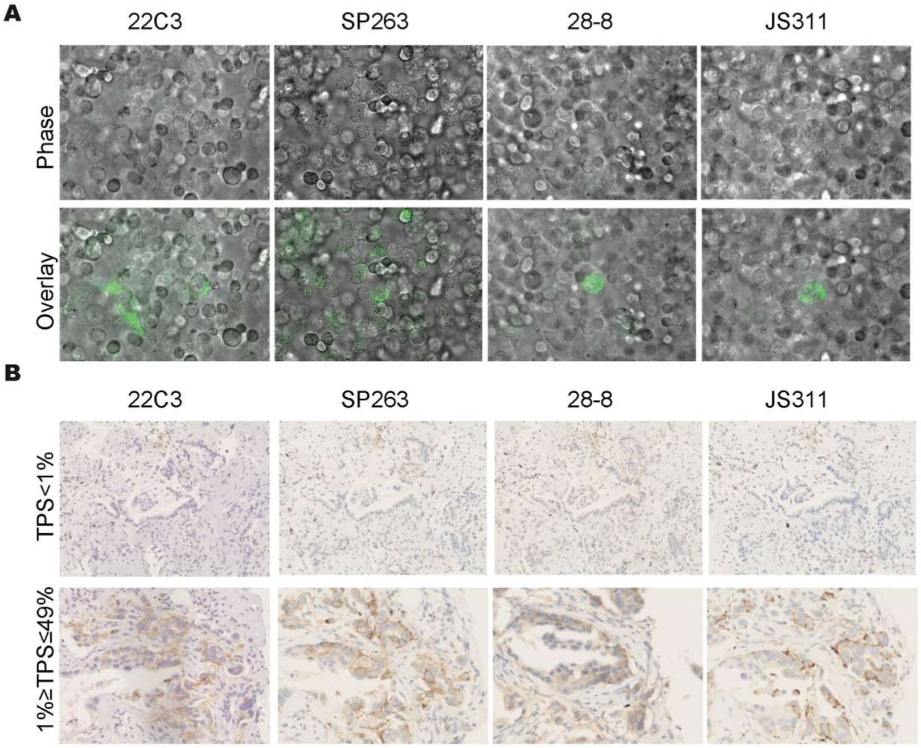

(A) The indirect immunofluorescence staining using the four PD-L1 antibodies. (B) The representative images of immunohistochemistry staining using four antibodies in three representative patients with PD-L1 TPS <1%, and 1≤ TPS ≤49%. (200x). PD-L1, programmed death receptor-ligand 1; TPS, tumor proportion score.

© 2020 Wang Z et al. *JAMA Network Open.*

eFigure 5. Flow Cytometry Analysis of 4 Antibodies in 5 Cell Lines

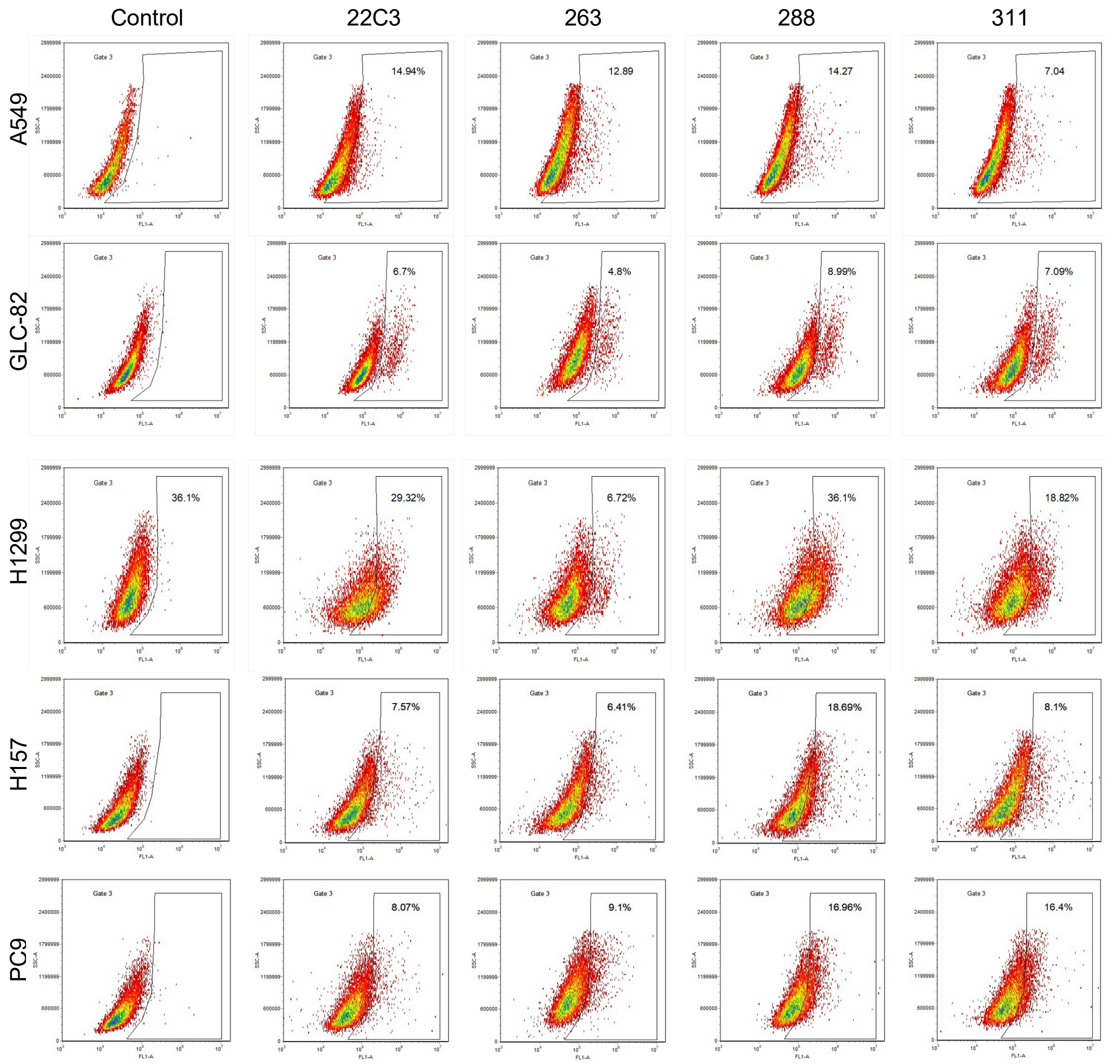

© 2020 Wang Z et al. *JAMA Network Open.*

eFigure 6. Indirect Immunofluorescence Staining Using 4 Antibodies in 5 Cell Lines

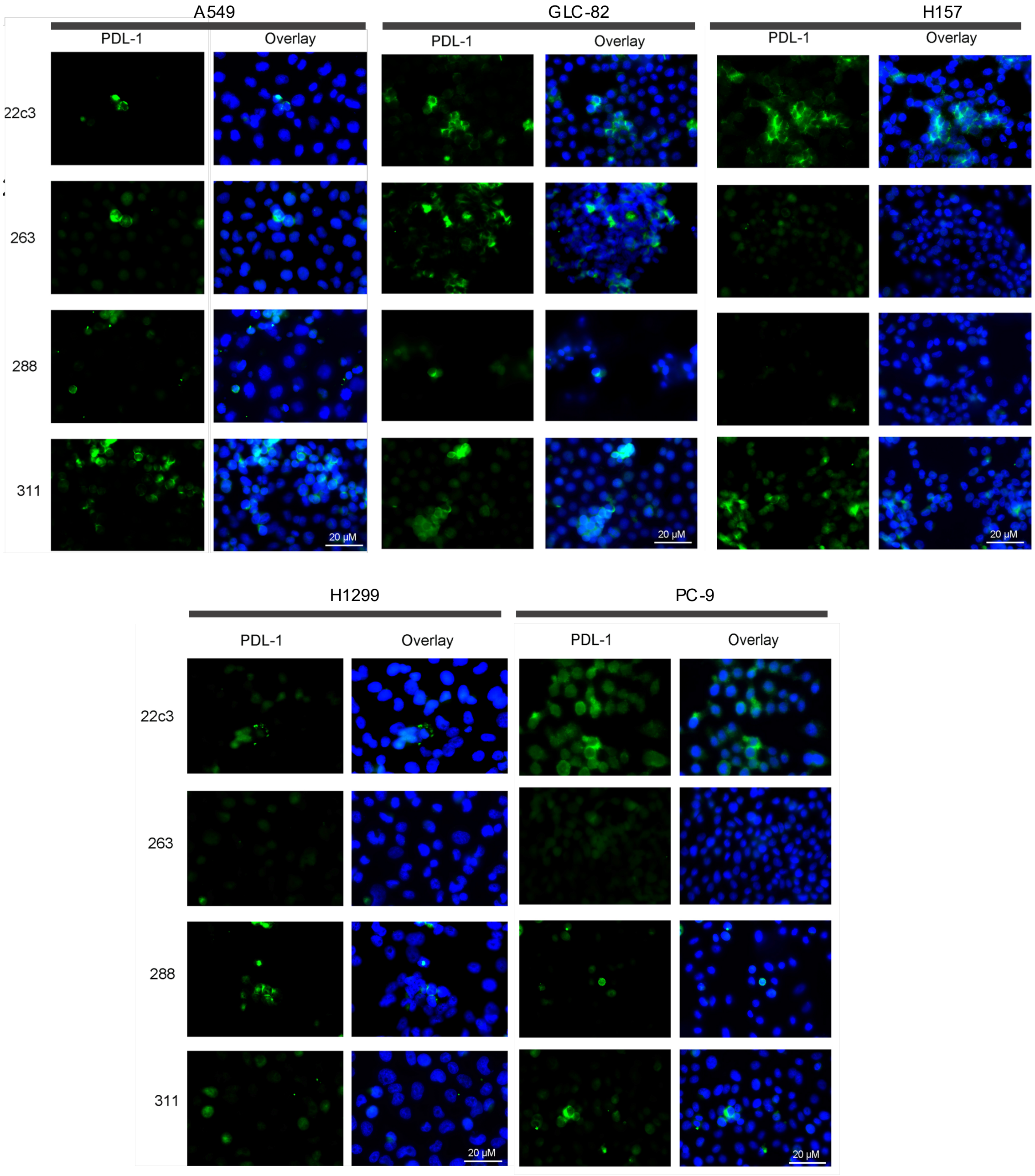

© 2020 Wang Z et al. *JAMA Network Open.*

eTable 1. Baseline Clinicopathological Characteristics

	Group A (N=20)	Group B (N=21)	Total (N=41)
**Age (years old)**			
Median (range)	59.5 (46 – 68)	57.0 (38 – 69)	59.0 (38 – 69)
**Gender**			
Female	5 (25.0)	7 (33.3)	12 (29.3)
Male	15 (75.0)	14 (66.7)	29 (70.7)
**ECOG score**			
0	7 (35.0)	6 (28.6)	13 (31.7)
1	13 (65.0)	15 (71.4)	28 (68.3)
**Pathology**			
Adenocarcinoma	13 (65.0)	16 (76.2)	29 (70.7)
Squamous carcinoma	6 (30.0)	4 (19.0)	10 (24.4)
Others	1 (5.0)	1 (4.8)	2 (4.9)
**Clinical Stage**			
IIIA	0	1 (4.8)	1 (2.4)
IIIB	1 (5.0)	2 (9.5)	3 (7.3)
IIIC	1 (5.0)	0	1 (2.4)
IV	18 (90.0)	18 (85.7)	36 (87.8)
**History of Surgery**			
Yes	9 (45.0)	5 (23.8)	14 (34.1)
No	11 (55.0)	16 (76.2)	27 (65.9)
**History of Radiotherapy**			
Yes	13 (65.0)	7 (33.3)	20 (48.8)
No	7 (35.0)	14 (66.7)	21 (51.2)
**History of Chemotherapy**			
Yes	20 (100.0)	21 (100.0)	41 (100.0)
No	0	0	0
**Lines of prior chemotherapy**			
1 line	8 (40.0)	9 (42.9)	17 (41.5)
2 lines	7 (35.0)	7 (33.3)	14 (34.1)
≥3 lines	5 (25.0)	5 (23.8)	10 (24.4)

© 2020 Wang Z et al. *JAMA Network Open.*

eTable 2. Pharmacokinetic Parameters of Patients

PK parameter	Unit	GroupA	Group B
		Mean ± SD (n=18)	Mean ± SD (n=17)
Kel	1/hr	0.003±0.001	0.004±0.004
$t_{1/2}$	hr	243.21±52.83	209.38±75.16
#T_{max}	hr	0.00~48.00	0.00~48.00
C_{max}	μg/ml	50.89(33.95~377.13)	63.51±10.78
$AUC_{(0-t)}$	hr*μg/ml	12465.28±4128.17	12331.42±2472.58
$AUC_{(0-inf)}$	hr*μg/ml	14844.76±4376.54	14384.25±3793.37
$AUC_{(t-inf)\%}$	%	16.31±6.69	12.75±6.92
Vd	ml/kg	75.80±23.21	61.67±14.00
Cl	ml/hr/kg	0.217±0.0554	0.226±0.0793
MRT_{inf}	hr	345.43±81.07	301.67±92.87

© 2020 Wang Z et al. *JAMA Network Open.*

eTable 3. Summary of Severe Adverse Events

Event, no. (%)	Group A (N=20)	Group B (N=21)	Total (N=41)
**Any Severe Adverse Events**	**3 (15.0%)**	**5 (23.8%)**	**8 (19.5%)**
Fever	1 (5.0%)	0	1 (2.4%)
Cancer pain	1 (5.0%)	0	1 (2.4%)
Interstitial lung disease	1 (5.0%)	1 (4.8%)	2 (4.9%)
Superior vein cava syndrome	0	1 (4.8%)	1 (2.4%)
Spinal cord compression	0	1 (4.8%)	1 (2.4%)
Disease progression	0	1 (4.8%)	1 (2.4%)
Death	0	1 (4.8%)	1 (2.4%)

© 2020 Wang Z et al. *JAMA Network Open.*

eTable 4. Best Response According to RECIST

n (%)	Group A (N=20)	Group B (N=21)	Total (N=41)
Best overall response （BOR）			
CR	0	0	
PR	0	2 (9.5%)	
SD	6 (30.0%)	3 (14.3%)	
PD	10 (50.0%)	7 (33.3%)	
NE	0	0	
Loss	4 (20.0%)	9 (42.9%)	
Objective response rate (ORR)	0	2 (9.5%)	2 (4.9%)
95% CI	(NA, NA)	(1.2, 30.4)	
Disease control rate (DCR)	6 (30.0%)	5 (23.8%)	11 (26.8%)
95% CI	(11.9, 54.3)	(8.2, 47.2)	

© 2020 Wang Z et al. *JAMA Network Open.*

eTable 5. Baseline Clinicopathological Characteristics of Assay Cohort

	N=280 (%)
**Age (years old)**	
Median (range)	60 (27– 86)
**Gender**	
Female	85 (30.4%)
Male	195 (69.6%)
**Pathology**	
Adenocarcinoma	168 (60.0%)
Squamous carcinoma	112 (40.0%)
**Clinical Stage**	
IA	23 (8.2%)
IB	15 (5.4%)
IIA	4 (1.4%)
IIB	22 (7.9%)
IIIA	27 (9.6%)
IIIB	25 (8.9%)
IIIC	8 (2.9%)
IVA	35 (12.5%)
IVB	121 (43.2%)
**Sample sources**	
Biopsy samples	277 (98.9%)
Surgical samples	3 (1.1%)
**Sample Loci**	
Lung	240 (85.7%)
Lymph nodes	28 (10.0%)
Subcutaneous nodules	5 (1.8%)
Liver	5 (1.8%)
Bone	1 (0.3%)
Peritoneum	1 (0.3%)

© 2020 Wang Z et al. *JAMA Network Open.*

eTable 6. Consistency Rate Among 4 Antibodies

	22C3		28-8		SP263		JS311	
	1% TPS	50% TPS	1% TPS	50% TPS	1% TPS	50% TPS	1% TPS	50% TPS
22C3			0.655 (82.8%)	0.691 (92.9%)	0.790 (89.5%)	0.742 (94.2%)	0.721 (85.4%)	0.688 (92.7%)
28-8	0.655 (82.8%)	0.691 (92.9%)			0.671 (83.5%)	0.693 (93.3%)	0.619 (80.8%)	0.684 (92.8%)
SP263	0.790 (89.5%)	0.742 (94.2%)	0.671 (83.5%)	0.693 (93.3%)			0.744 (86.6%)	0.773 (94.9%)
JS311	0.721 (85.4%)	0.688 (92.7%)	0.619 (80.8%)	0.684 (92.8%)	0.744 (86.6%)	0.773 (94.9%)		

© 2020 Wang Z et al. *JAMA Network Open.*

eTable 7. Consistency Rate Among 4 Antibodies in Lung Adenocarcinoma

	22C3		28-8		SP263		RM311	
	1% TPS	50% TPS	1% TPS	50% TPS	1% TPS	50% TPS	1% TPS	50% TPS
22C3			0.694 (85.0%)	0.774 (94.4%)	0.791 (89.7%)	0.831 (96.4%)	0.722 (85.9%)	0.716 (93.9%)
28-8	0.694 (85.0%)	0.774 (94.4%)			0.633 (81.9%)	0.773 (94.4%)	0.615 (81.0%)	0.711 (93.0%)
SP263	0.791 (89.7%)	0.831 (96.4%)	0.633 (81.9%)	0.773 (94.4%)			0.742 (86.7%)	0.829 (96.4%)
RM311	0.722 (85.9%)	0.716 (93.9%)	0.615 (81.0%)	0.711 (93.0%)	0.742 (86.7%)	0.829 (96.4%)		

© 2020 Wang Z et al. *JAMA Network Open.*

eTable 8. Consistent Rate Among 4 Antibodies in Squamous Cell Carcinoma

	22C3		28-8		SP263		RM311	
	1% TPS	50% TPS	1% TPS	50% TPS	1% TPS	50% TPS	1% TPS	50% TPS
22C3			0.588 (79.4%)	0.572 (90.7%)	0.785 (89.3%)	0.624 (91.1%)	0.710 (84.7%)	0.653 (91.0%)
28-8	0.588 (79.4%)	0.572 (90.7%)			0.719 (86.0%)	0.562 (91.6%)	0.614 (80.4%)	0.661 (92.5%)
SP263	0.785 (89.3%)	0.624 (91.1%)	0.719 (86.0%)	0.562 (91.6%)			0.741 (86.5%)	0.701 (92.8%)
RM311	0.710 (84.7%)	0.653 (91.0%)	0.614 (80.4%)	0.661 (92.5%)	0.741 (86.5%)	0.701 (92.8%)		

© 2020 Wang Z et al. *JAMA Network Open.*

eReferences

[1] Yang M, Liu J, Wang F et al. Lysyl oxidase assists tumorinitiating cells to enhance angiogenesis in hepatocellular carcinoma. Int J Oncol 2019; 54: 1398-1408.

[2] Xu XL, Xing BC, Han HB et al. The properties of tumor-initiating cells from a hepatocellular carcinoma patient's primary and recurrent tumor. Carcinogenesis 2010; 31: 167-174.

© 2020 Wang Z et al. *JAMA Network Open.*
